# Immunotherapy-Based Conversion to Curative-Intent Treatment in Hepatocellular Carcinoma: A Multidisciplinary Framework

**DOI:** 10.3390/cancers18142234

**Published:** 2026-07-12

**Authors:** Kizuki Yuza, Timothy M. Pawlik

**Affiliations:** 1 Department of Surgery, The Ohio State University Wexner Medical Center and James Comprehensive Cancer Center, Columbus, OH 43210, USA; kizuki.yuza@osumc.edu; 2 Department of Gastroenterological Surgery, Yokohama City University, Yokohama 236-0004, Japan

**Keywords:** hepatocellular carcinoma, immunotherapy, conversion therapy, downstaging, curative-intent treatment, liver resection, liver transplantation, multidisciplinary care

## Abstract

Immune checkpoint inhibitor (ICI)-based combinations have become central systemic treatment options for advanced hepatocellular carcinoma (HCC) and are now being integrated into selected intermediate-stage settings. As tumor responses have improved, some patients who were not initially candidates for curative-intent treatment may later become candidates for resection, ablation, or liver transplantation. However, radiographic response alone does not define curative-intent candidacy, and no shared framework currently guides how post-immunotherapy response should be translated into a treatment decision. Terminology also differs regionally: Asian literature frames a resection-anchored paradigm, whereas Western practice uses transplant-anchored downstaging. This narrative review proposes a multidisciplinary framework for immunotherapy-based conversion to curative-intent treatment in HCC. We first clarify the lexicon of conversion, downstaging, bridging, neoadjuvant therapy, post-ICI transplantation, and drug-free or treatment-free status. We then summarize conversion-relevant evidence across key clinical decision settings, including transarterial chemoembolization (TACE)-unsuitable intermediate-stage disease, portal vein tumor thrombus or macrovascular invasion, borderline-resectable or locally advanced disease, and transplant downstaging or bridging. The central framework defines curative-intent transition through the intersection of three domains: technical suitability, oncologic suitability, and physiologic or liver-reserve suitability. Biomarkers, imaging response, tumor-marker kinetics, liver function, and treatment-related toxicity are discussed as inputs into candidacy rather than as response measures alone. Finally, we propose a multidisciplinary workflow and highlight lessons from pancreatic cancer, biliary tract cancer, and colorectal liver metastases. As an expert-opinion-based framework, this approach should structure multidisciplinary discussion rather than serve as validated selection criteria; harmonized terminology, prospective conversion registries, and conversion-specific endpoints are needed for prospective validation.

## Simple Summary

Hepatocellular carcinoma, a common type of liver cancer, is a major cause of cancer-related death worldwide. Surgery, ablation, and liver transplantation can offer long-term control or cure, but many patients are not eligible for these treatments when the cancer is first diagnosed. New immunotherapy-based treatments can shrink tumors in some patients, creating a chance to reconsider treatments intended to provide long-term control or cure. Tumor shrinkage alone is not enough, however, to decide whether surgery, ablation, or transplantation is appropriate. In this review, we propose a practical framework for making this decision. The framework asks three questions: whether the treatment is technically possible, whether the cancer biology supports long-term benefit, and whether the patient’s liver and overall condition can tolerate the planned treatment. We also propose a multidisciplinary workflow to guide these decisions after immunotherapy.

## 1. Introduction

Hepatocellular carcinoma (HCC) remains a leading cause of cancer-related mortality worldwide [1]. The epidemiology of HCC is also changing. Although viral hepatitis is increasingly controlled in many regions, metabolic risk factors are rising and now contribute substantially to the global burden of HCC [2]. Curative-intent treatment is confined to resection, ablation, or liver transplantation, and is appropriate only for selected patients with adequate liver function, manageable tumor burden, and acceptable operative risk. Many patients, however, are not candidates for curative-intent treatment at presentation because of tumor extent, transplant ineligibility, impaired liver reserve, or physiologic risk.

Systemic therapy for advanced HCC has changed over the past two decades. Sorafenib and lenvatinib first established tyrosine kinase inhibitors as the initial systemic standard [3,4]. Later-line tyrosine kinase inhibitors, including regorafenib after sorafenib progression, expanded options for selected patients [5]. Beginning in 2020, immune checkpoint inhibitor (ICI)-based combinations became central first-line options, building on earlier clinical experience with ICI therapy in unresectable HCC [6]. Atezolizumab plus bevacizumab and tremelimumab plus durvalumab positioned ICI-based therapies as important treatment options for advanced HCC [7,8]. Subsequent phase 3 trials of nivolumab plus ipilimumab and camrelizumab plus rivoceranib further expanded the first-line landscape [9,10]. At the same time, not all ICI-based combinations have improved overall survival [11,12]. Phase 3 evidence has also extended ICI-based combinations into intermediate-stage, transarterial chemoembolization (TACE)-eligible disease [13,14]. The broader immunotherapy landscape and its current frontiers have been comprehensively reviewed elsewhere [15]. Importantly, these trials were designed to evaluate systemic disease control, not to define when response should trigger transition to resection, ablation, or transplantation.

As systemic options have expanded, a new clinical question has emerged. Some patients who were not initially candidates for curative-intent treatment now achieve sufficient response to systemic therapy that resection, ablation, or transplantation can be reconsidered. Asian centers have commonly framed this pathway as conversion therapy [16,17,18]. Western literature, by contrast, has often used the language of downstaging within transplant-anchored selection frameworks [19]. This terminology divergence reflects a deeper conceptual gap. Radiographic response alone is not a candidacy criterion for curative-intent surgery, ablation, or transplantation. Yet, no shared multidisciplinary framework currently defines how post-immunotherapy response should be translated into a curative-intent treatment decision.

This review proposes a multidisciplinary framework for immunotherapy-based conversion to curative-intent treatment in HCC. The central premise is that curative-intent transition after immunotherapy should be defined by the intersection of three domains: technical suitability, oncologic suitability, and physiologic or liver-reserve suitability. This multidimensional assessment should be structured through a multidisciplinary team workflow. The review first establishes a shared lexicon for clinical decision-making (Section 2), summarizes conversion-relevant evidence by clinical decision setting (Section 3), defines the three-domain candidacy framework (Section 4), reviews biomarkers that may inform candidacy rather than response alone (Section 5), proposes a multidisciplinary workflow (Section 6), and draws comparative lessons from adjacent gastrointestinal oncology fields (Section 7).

This review provides a targeted synthesis of the literature rather than a systematic review or meta-analysis. Relevant English-language reports were identified in PubMed/MEDLINE through June 2026, supplemented by manual review of key references. Priority was given to phase 3 randomized trials, prospective phase 2 studies, clinical practice guidelines, and expert consensus statements addressing immunotherapy-based systemic therapy, conversion therapy, downstaging, neoadjuvant treatment, and post-immunotherapy curative-intent treatment in HCC. Retrospective series and single-arm conversion cohorts were included when these studies informed emerging clinical decision points, but were interpreted as lower-certainty, hypothesis-generating evidence. Particular attention was paid to whether studies specified baseline candidacy, treatment sequence, response criteria, the final curative-intent treatment delivered, and post-treatment outcomes. No quantitative synthesis or formal risk-of-bias scoring was performed; evidence certainty was instead assessed qualitatively from study design, endpoint relevance, population generalizability, and susceptibility to selection or publication bias.

## 2. Terminology and Regional Paradigms in Curative-Intent Transition

The terminology used to describe movement from non-curative-intent HCC to resection, ablation, or transplantation has evolved rapidly, but definitions have not kept pace. Conversion therapy, downstaging, bridging therapy, and neoadjuvant therapy are often used interchangeably in clinical discussion and in the literature, although they differ in baseline candidacy, intended curative endpoint, and regional practice context. This ambiguity matters. It complicates trial design, limits cross-regional comparison, and can obscure decision-making within multidisciplinary teams. A shared working lexicon is therefore needed before post-treatment candidacy can be assessed in a consistent way.

### 2.1. Conversion, Downstaging, Bridging, and Neoadjuvant Therapy

In this review, conversion therapy refers to systemic and/or locoregional treatment intended to render initially non-curative-intent HCC eligible for resection, ablation, or transplantation, following the Chinese expert consensus on conversion therapy and subsequent Asian consensus statements [16,17,18]. The term is used most often in Asian resection-anchored practice, although it is increasingly appearing in Western literature. Downstaging, as used in Western transplant-anchored practice, refers specifically to reducing tumor burden from beyond predefined transplant criteria to within acceptable limits for liver transplantation [19]. It is usually applied within protocol-defined transplant frameworks such as Milan, University of California San Francisco (UCSF), United Network for Organ Sharing (UNOS) downstaging, or alpha-fetoprotein (AFP)-based models [20,21,22]. Bridging therapy is given to prevent tumor progression while a patient who already meets accepted criteria awaits transplantation; its goal is to maintain eligibility rather than to restore it [19,23]. Neoadjuvant therapy applies to patients who are already candidates for curative-intent treatment at baseline and is administered before planned resection or transplantation to define tumor biology, treat micro-metastatic disease, and potentially improve recurrence-free or overall survival [24,25,26,27].

Two additional terms have become important in the immunotherapy era. Post-ICI transplantation, or ICI bridge to transplantation, refers to ICI-based therapy before liver transplantation, followed by reassessment of transplant candidacy, washout interval, and rejection risk [23,28,29]. Drug-free or treatment-free status describes sustained complete or near-complete response after discontinuation of systemic therapy in patients who do not undergo local curative treatment [30]. Neither concept should be assumed to represent anatomic cure. Table 1 summarizes these terms according to typical starting scenario, intended endpoint, and common pitfalls.

### 2.2. Western Transplant-Anchored Paradigm

Western HCC management has historically been shaped by liver transplantation. The Milan criteria established the dominant eligibility framework in 1996 [31], and subsequent models refined transplant selection and post-transplant recurrence prediction [20,21,22,32]. UNOS downstaging protocols and successive American Association for the Study of Liver Diseases practice guidance documents then incorporated these concepts into waitlist-based selection pathways [19,33]. Liver-reserve considerations, particularly Model for End-Stage Liver Disease (MELD) score and clinically significant portal hypertension, remain closely linked to transplant decision-making, while donor availability limits how broadly conversion can be pursued. Within this setting, prior ICI exposure has introduced a new sequencing problem: how to translate post-ICI response into safe transplant timing without increasing the risk of allograft rejection [23,28,29].

### 2.3. Asian Resection-Anchored Paradigm

Asian centers, by contrast, have generally placed greater emphasis on resection. Hepatitis B-driven disease, higher baseline tumor burden, broader acceptance of resection for portal vein tumor thrombus (PVTT), and limited access to transplantation in some settings have produced a literature in which conversion therapy usually denotes treatment intended to enable hepatectomy [16,17,18]. This surgical orientation is supported by Japanese nationwide data suggesting that, in selected patients with portal vein invasion, liver resection may confer a survival benefit over non-surgical treatment when tumor thrombus is limited to a first-order portal branch (Vp3) [34]. Asia-Pacific consensus statements have formalized TACE-unsuitable intermediate-stage HCC and positioned systemic therapy, often integrated with locoregional treatment, as the preferred first-line strategy for this subgroup, while regional guidance has clarified the sequencing of TACE and systemic therapy [35,36,37]. Drug-free or treatment-free status after atezolizumab plus bevacizumab has also been discussed within this practice context as a potential endpoint for patients who are unsuitable for local therapy [30].

### 2.4. Need for a Shared Multidisciplinary Lexicon

The Western and Asian paradigms share the same broad intent: to preserve, restore, or create an opportunity for curative-intent treatment. These paradigms differ, however, in the dominant endpoint and in how success is defined. A patient treated with systemic therapy followed by hepatectomy in an Asian center and a patient treated with systemic therapy followed by transplantation in a Western center may both be described as having undergone conversion or downstaging, even though the clinical assumptions, response criteria, and reported outcomes are not directly comparable. ICI combinations have made this problem more visible by creating new candidate populations and new sequencing constraints. For this reason, reports of immunotherapy-based conversion should specify the baseline status, intended curative endpoint, treatment sequence, response criteria, and final treatment delivered. This shared vocabulary provides the basis for the candidacy framework and multidisciplinary workflow proposed in Section 4 and Section 6.

## 3. Current Evidence for Immunotherapy-Based Conversion Strategies

First-line systemic therapy for advanced HCC now includes four positive phase 3 immunotherapy-based regimens: atezolizumab plus bevacizumab, tremelimumab plus durvalumab, nivolumab plus ipilimumab, and camrelizumab plus rivoceranib [7,8,9,10]. Two additional ICI-tyrosine kinase inhibitor combinations did not improve overall survival [11,12]. Phase 3 evidence has also validated TACE plus immunotherapy in TACE-eligible intermediate-stage disease [13,14]. Across these settings, however, conversion has rarely been studied as a primary endpoint. Most trials were designed around progression-free or overall survival, while transition to resection, ablation, or transplantation appears mainly in subgroup analyses, retrospective series, or prospective single-arm cohorts; recent commentary has likewise emphasized that conversion to resection or transplantation remains undefined and unmeasured as a formal endpoint in first-line immunotherapy trials [38]. The sections below therefore organize the evidence by clinical decision setting rather than by regimen. Trial-level design, population, and efficacy details are summarized in Table 2 and Supplementary Table S1.

The evidence was interpreted hierarchically. Phase 3 randomized trials were regarded as high-certainty evidence for systemic disease control within their enrolled populations, but as lower-certainty evidence for conversion decision-making because transition to resection, ablation, or transplantation was not a prespecified endpoint. Prospective single-arm conversion studies were considered informative for feasibility but limited by patient selection and the absence of randomized comparators. Retrospective cohorts were treated as hypothesis-generating, given their susceptibility to selection and publication bias. Table 2 therefore distinguishes evidence certainty for conversion decisions from the certainty of systemic disease-control benefit.

### 3.1. TACE-Unsuitable Intermediate-Stage HCC

The Asia-Pacific Primary Liver Cancer Expert consensus formalized “TACE-unsuitable intermediate-stage HCC” as a clinical category in 2020, recognizing that not all Barcelona Clinic Liver Cancer (BCLC)-B patients benefit from repeated TACE and that systemic integration may be a more appropriate first-line strategy [35]. TACE may also induce immunogenic changes in the tumor microenvironment, providing a biological rationale for combining locoregional therapy with immunotherapy [48]. Two phase 3 trials have moved this approach into the intermediate-stage setting: EMERALD-1 and LEAP-012 each prolonged progression-free survival when systemic therapy was integrated with TACE [13,14]. A recent commentary framed these trials as redefining the treatment standard for selected BCLC-B patients rather than simply validating TACE alone [39].

For patients who are TACE-unsuitable from the outset—such as individuals with bilobar high-burden disease, infiltrative tumors, or impaired liver reserve—early-phase Asian studies have reported surgical conversion after ICI-based combination therapy in selected responders, including R0 resection after envafolimab plus lenvatinib with TACE [40]. A separate endpoint in this population is sustained drug-free or treatment-free status after atezolizumab plus bevacizumab, observed in approximately one-fifth of treated patients [30]. Treatment-free disease control should not be equated with anatomic cure, but it has become a relevant endpoint when local curative treatment is not feasible. In this setting, the conversion question is whether tumor control can be translated into a safe local intervention, or whether durable treatment-free control is the more appropriate goal.

### 3.2. Macrovascular Invasion and Portal Vein Tumor Thrombus

Macrovascular invasion, particularly Vp3 and Vp4 PVTT, has long limited curative-intent treatment. The FOHAIC-1 phase 3 trial reported that hepatic arterial infusion chemotherapy (HAIC) improved overall survival compared with sorafenib in advanced HCC with predominantly Vp3 or Vp4 disease, with subsequent curative surgery or ablation reported in a downstaged subset [41]. Radiation-based integration is supported by RTOG 1112, in which stereotactic body radiotherapy (SBRT) plus sorafenib improved overall survival compared with sorafenib in advanced HCC that included patients with macrovascular invasion [43]. Smaller phase 2 data with SBRT plus pembrolizumab have shown a response signal in technically challenging anatomy, although phase 3 confirmation is pending [44]. Yttrium-90 radioembolization with personalized dosimetry has also improved tumor response in selected patients with locally advanced HCC, including portal vein invasion [49].

Preoperative tyrosine kinase inhibitor-based strategies have also been tested as conversion approaches. The LENS-HCC multicenter phase 2 study of preoperative lenvatinib supported surgical feasibility in selected patients [45]. A separate multicenter retrospective study of large HCC with major PVTT noted that adding HAIC to lenvatinib plus drug-eluting bead TACE improved response and survival compared with the same regimen without HAIC [42]. More direct prospective evidence comes from the single-center SILENSES phase 2 expansion trial, in which sintilimab plus lenvatinib was evaluated with conversion as a primary endpoint in initially unresectable, predominantly BCLC-C disease; however, its single-arm design and predominantly hepatitis B-related Asian population limit generalizability [46]. Together, these data suggest that PVTT should not be regarded as an automatic barrier to curative-intent transition in selected patients.

These findings nevertheless require careful interpretation. Most conversion cohorts in patients with macrovascular invasion or advanced-stage HCC comprise highly selected responders who maintained adequate liver function, performance status, and procedural candidacy during systemic or multimodal therapy. Patients who progressed early, developed treatment-related toxicity, or lost liver reserve are therefore underrepresented in surgical conversion cohorts, creating responder, referral, and immortal-time bias. Many reports also originate from Asian, hepatitis B virus-predominant populations and from centers with substantial expertise in hepatectomy for portal vein tumor thrombus, hepatic arterial infusion chemotherapy, or complex multimodal treatment. Favorable post-resection outcomes in these series should not be interpreted as demonstrating that conversion surgery itself improves survival relative to continued systemic therapy or other non-surgical strategies, and publication bias may further inflate the apparent benefit of conversion. Immunotherapy-specific phase 3 trials with conversion endpoints are also lacking. For patients with macrovascular invasion, the key question is therefore not simply whether the thrombus has regressed radiographically, but whether vascular response is durable, extrahepatic spread is absent, liver reserve is preserved, and a technically coherent resection or ablation plan exists.

### 3.3. Borderline-Resectable or Locally Advanced HCC

Borderline-resectable HCC—disease that is anatomically resectable but at high risk for early recurrence or technically demanding to resect—has emerged as a natural setting for neoadjuvant immunotherapy, paralleling the borderline-resectable framework established in pancreatic adenocarcinoma. Three single-arm phase 2 studies form the early evidence base. Marron and colleagues treated resectable HCC with neoadjuvant single-agent cemiplimab and reported a major pathologic response in 20 percent of patients [26]. Kaseb and colleagues compared perioperative nivolumab alone with nivolumab plus ipilimumab, with major or complete pathologic responses observed in both arms [25]. Ho and colleagues administered preoperative cabozantinib plus nivolumab in locally advanced HCC, providing proof of concept for conversion to R0 resection in a small cohort [24]. Long-term efficacy and biomarker data from a perioperative ICI cohort have since extended these observations [47].

Two practical issues complicate interpretation. First, pathologic response after ICI-based therapy may be more informative than radiographic response when selecting patients for resection [27]. In a cross-trial, patient-level analysis of neoadjuvant ICI therapy before liver resection, major pathologic response was associated with significantly longer relapse-free survival, and nearly one-third of major pathologic responses were not predicted by radiographic response [50]. Second, the optimal duration of preoperative therapy and the role of adjuvant ICI after conversion surgery remain unsettled; the adjuvant question is discussed in Section 6.4, with reference to the IMbrave050 trial and its updated overall survival analysis [51,52]. Using this evidence base, neoadjuvant ICI in borderline-resectable HCC is best viewed not only as cytoreduction, but also as a way to test tumor biology before committing to curative-intent treatment. Consensus criteria are still needed to translate radiographic, pathologic, and biomarker response into reproducible treatment decisions.

### 3.4. Transplant Downstaging and Bridging

Liver transplantation after ICI exposure has moved from a theoretical safety concern to an emerging clinical pathway. The multicenter VITALITY study suggested that transplantation after ICI bridging or downstaging is feasible in selected waitlisted patients [23]. Retrospective and pooled analyses indicate overall rejection rates of approximately 15 to 20 percent, with longer washout intervals, particularly beyond 50 days, associated with lower rejection risk [28,29]. These data support feasibility in selected patients while underscoring the need for careful attention to washout interval, prior immune-related adverse events (irAEs), and immunosuppression strategy.

Non-immunotherapy bridging modalities may also expand the technical options available to maintain or restore transplant eligibility, as illustrated by the histotripsy pivotal trial in selected anatomy [53]. Across this pathway, ICI exposure changes the safety and timing calculus of transplantation more than the underlying tumor burden criteria themselves. Candidacy remains anchored to protocol-defined transplant frameworks, with additional reassessment of immune washout and rejection risk. These sequencing considerations are addressed in the multidisciplinary workflow proposed in Section 6.

## 4. Defining Curative-Intent Candidacy After Immunotherapy

**Figure 1:**
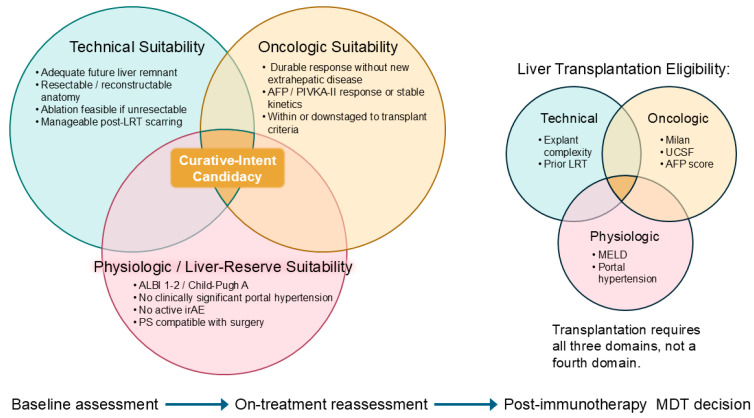
Proposed three-domain framework for curative-intent candidacy after immunotherapy-based conversion in hepatocellular carcinoma. Curative-intent transition after immunotherapy requires reassessment of three intersecting domains: technical suitability, oncologic suitability, and physiologic or liver-reserve suitability. Liver transplantation is integrated within the same framework as a curative-intent pathway requiring all three domains, rather than as a separate fourth domain. Representative considerations are shown for illustration; detailed domain-specific considerations are discussed in Section 4. The arrows along the bottom indicate the sequential progression from baseline assessment through on-treatment reassessment to the post-immunotherapy multidisciplinary team decision. The framework is adapted from the anatomic, biologic, and conditional triad established for borderline-resectable pancreatic adenocarcinoma. This proposed framework is intended to support multidisciplinary discussion and should not be interpreted as validated clinical criteria. Abbreviations: AFP, alpha-fetoprotein; ALBI, albumin-bilirubin; irAE, immune-related adverse event; LRT, locoregional therapy; MDT, multidisciplinary team; MELD, Model for End-Stage Liver Disease; PIVKA-II, protein induced by vitamin K absence or antagonist-II; PS, performance status; UCSF, University of California San Francisco.

Response after ICI-based therapy is necessary but not sufficient to convert a patient with previously non-curative-intent HCC into a candidate for resection, ablation, or transplantation. Radiographic improvement, even when durable, does not by itself establish that an operation is technically feasible, that the disease biology supports curative-intent treatment, or that the patient can tolerate the intended procedure. Curative-intent transition therefore requires reassessment across three intersecting dimensions: technical suitability, oncologic suitability, and physiologic or liver-reserve suitability. Figure 1 summarizes this proposed framework, which is intended to structure multidisciplinary discussion and future study design rather than to serve as validated eligibility criteria.

### 4.1. Rationale for a Three-Domain Candidacy Framework

Multidomain candidacy assessment has precedent in gastrointestinal surgical oncology. Borderline-resectable pancreatic adenocarcinoma was defined in 2008 along three axes: anatomic resectability, biologic uncertainty, including suspicious metastatic findings or aggressive markers, and conditional fitness, including performance status and comorbidity [54]. The National Comprehensive Cancer Network guideline formalized this triad into clinical practice [55], and the PREOPANC randomized trial supported neoadjuvant treatment in this population, improving disease-free survival and the R0 resection rate compared with upfront surgery, with a long-term overall survival benefit [56,57]. This construct has since shaped pancreatic surgical selection.

Applying a similar concept to HCC requires disease-specific adaptation. In HCC, resectability is constrained not only by tumor anatomy and biology, but also by underlying liver disease, transplant eligibility, and treatment-specific physiologic risk. Cirrhosis, clinically significant portal hypertension, prior locoregional therapy, immune-related hepatitis, and anti-vascular endothelial growth factor perioperative risk all influence whether a patient who has responded to ICI-based therapy can safely proceed to curative-intent treatment. An international multicenter cohort recently validated a borderline-resectability construct specific to HCC, using tumor burden score within a multidisciplinary framework to stratify survival after resection [58]. The 2026 BCLC similarly recognizes that major response after immunotherapy creates a distinct post-treatment state—termed therapeutic conversion—rather than a return to the original staging category, reinforcing the need for a response-specific candidacy framework [59]. In this review, post-conversion candidacy is therefore conceptualized as the intersection of three suitability domains, as outlined in Figure 1.

### 4.2. Technical Suitability

Technical suitability asks whether the intended curative treatment is anatomically and procedurally feasible after ICI-based therapy. For resection, this assessment includes the location and number of residual viable lesions, their relationship to major vascular structures, the adequacy of the future liver remnant, and the feasibility of vascular reconstruction when needed. For selected patients, ablation may provide an alternative curative-intent endpoint when resection is not feasible, but the disease remains oncologically appropriate and physiologically tolerable.

Prior locoregional therapy can substantially alter the technical assessment. TACE, HAIC, and SBRT may produce parenchymal inflammation, capsular adhesions, or distorted tissue planes. ICI-related immune infiltration may also alter operative findings, although reproducible histologic patterns have not been established. Technical reassessment is therefore best performed on updated cross-sectional imaging close to the time of planned intervention, with input from hepatobiliary surgery, interventional radiology, and transplant surgery when relevant. Reference standards for major hepatectomy outcomes, including global benchmarks for complication-free recovery, provide useful context when estimating post-conversion operative risk [60].

### 4.3. Oncologic Suitability

Oncologic suitability asks whether tumor behavior supports a curative-intent approach. The relevant question is not only whether the tumor has decreased in size, but whether the response appears durable and biologically meaningful. Sustained radiographic response over several months provides more reassurance than a single favorable scan. Composite measures of tumor burden, such as the tumor burden score, integrate lesion number and largest diameter and have been validated as prognostic stratifiers after HCC resection [61,62]. Tumor-marker kinetics, particularly serial changes in AFP and protein induced by vitamin K absence or antagonist-II (PIVKA-II), add a biologic dimension that imaging alone cannot fully capture; biomarker assessment is discussed in Section 5.

The absence of new extrahepatic disease during treatment remains a baseline requirement. Conversely, several findings should prompt caution despite apparent radiographic response, including short response duration, any new extrahepatic lesion, rising AFP despite radiographic stability, infiltrative growth pattern, or persistent viable tumor within a portal vein tumor thrombus. For patients being considered for liver transplantation, post-treatment disease should be within or downstaged to Milan, UCSF, Metroticket 2.0, or AFP-based transplant criteria [20,22,31,32]. The RETREAT score further quantifies post-transplant recurrence risk in patients downstaged to within criteria [21]. None of these tools, however, were developed in the immunotherapy era, and their applicability to patients with deep ICI-induced responses requires prospective validation.

### 4.4. Physiologic and Liver-Reserve Suitability

Physiologic suitability asks whether the patient can tolerate the intended treatment and, in the case of transplantation, the perioperative immunosuppressive course. Liver reserve is the central constraint. The albumin-bilirubin (ALBI) grade provides an evidence-based measure of liver function in HCC and offers finer granularity than the Child–Pugh classification, particularly among Child–Pugh A patients [63,64,65]. Dynamic ALBI trajectories during treatment, including paired changes with the fibrosis-4 index, have also been proposed as predictors of post-resection complication risk [66]. Preserved Child–Pugh A status with ALBI grade 1 or 2 generally supports curative-intent treatment, whereas clinically significant portal hypertension, prior hepatic decompensation, or active immune-related hepatitis should prompt caution.

Body composition adds another layer to physiologic assessment. Sarcopenia, assessed by skeletal muscle index on cross-sectional imaging, has been associated with worse short- and long-term outcomes after hepatectomy for HCC in independent European cohorts [67,68]. Hepatic decompensation is particularly important in the immunotherapy era. In a large real-world cohort, hepatic decompensation was the major driver of mortality among patients treated with atezolizumab plus bevacizumab, carrying a higher mortality risk than tumor progression [69]. A parallel real-world cohort confirmed liver decompensation as a frequent and prognostically dominant event in HCC patients receiving first-line systemic immunotherapy [70], and hepatic decompensation has been proposed as a distinct clinical outcome that should be reported alongside tumor progression in HCC trials [71]. The CheckMate 9DW phase 3 trial of nivolumab plus ipilimumab reported an early hazard ratio for death of 1.65 within the first six months of randomization before the longer-term survival benefit emerged [9]. These observations reinforce the need to assess physiologic suitability before and during ICI-based therapy; specifically, patients with marginal liver reserve may not survive long enough to benefit from durable immunologic disease control.

### 4.5. Liver Transplantation Within the Three-Domain Framework

Liver transplantation is a distinct curative-intent pathway, but it is best understood within the same three-domain framework rather than as a separate fourth domain. Milan, UCSF, and AFP-based criteria belong primarily to the oncologic domain; MELD score, clinically significant portal hypertension, and decompensation history belong to the physiologic domain; and explant complexity, prior locoregional therapy, and biliary anatomy belong to the technical domain. The RETREAT score, which incorporates post-downstaging AFP, microvascular invasion on explant, and largest viable tumor diameter, illustrates how multidomain inputs are already integrated in transplant selection [21]. Section 3.4 and Section 6 discuss the additional sequencing considerations introduced by pre-transplant ICI exposure, including washout interval and irAE history. These considerations modify the transplant pathway, but they do not replace the three-domain assessment.

## 5. Biomarkers for Conversion Candidacy Rather Than Response Alone

The biomarker question in immunotherapy-based conversion is different from the biomarker question in advanced HCC. In advanced disease, the main question is whether the tumor is likely to respond. In the conversion setting, the question is whether response should lead to resection, ablation, or transplantation. The same biological information is therefore interpreted against a different clinical threshold. A useful conversion biomarker should inform not only response, but also the technical, oncologic, and physiologic dimensions described in Section 4. The markers discussed below are grouped according to the domain they most directly inform: tumor-marker kinetics and tissue-based signals mainly inform oncologic suitability, imaging response informs both technical and oncologic suitability, and liver-reserve measures inform physiologic suitability.

### 5.1. Tumor-Marker Kinetics

AFP and PIVKA-II remain the most widely used serum tumor markers in HCC. During systemic therapy, serial kinetics are usually more informative than baseline values alone. A substantial AFP decline or normalization after ICI-based treatment may support oncologic candidacy for resection or transplantation, and AFP-based models such as the AFP-French model already incorporate post-treatment AFP into transplant eligibility [22]. PIVKA-II is used more commonly in Asian conversion practice, and its combination with tumor burden score has been proposed to refine candidacy assessment, particularly for AFP-negative HCC [72]. Discordance between marker kinetics and radiologic response is common after immunotherapy. Such discordance should prompt multidisciplinary reassessment rather than automatic action based on either signal alone.

### 5.2. Imaging and Radiologic–Pathologic Discordance

Modified RECIST (mRECIST), which was developed to capture viable tumor through arterial-phase enhancement, remains the dominant radiologic response framework in HCC trials [73]. The Liver Imaging Reporting and Data System Treatment Response (LI-RADS TR) algorithm provides a standardized lexicon—viable, equivocal, nonviable, or nonevaluable—for lesions treated with locoregional therapy [74]. Both frameworks predate the immunotherapy era and have not been fully validated for ICI-induced response patterns.

Radiologic–pathologic discordance is a central problem in conversion decision-making. Viable tumors may be present on explant pathology despite radiologic complete response, whereas persistent enhancement may reflect inflammatory infiltrate rather than viable disease. Discrepancies between imaging-based and pathologic tumor size measurements have been documented in HCC resection specimens [75]. Consistent with this, a cross-trial analysis of neoadjuvant ICI therapy found that radiographic response only partially captured pathologic response, with a clinically meaningful subset of major pathologic responses missed by imaging-based criteria [50]. For conversion candidacy, residual enhancement on mRECIST or LI-RADS TR assessment should therefore be interpreted in clinical context. Depending on anatomy, liver reserve, and treatment trajectory, the appropriate response may be continued therapy, short-interval repeat imaging, selective tissue confirmation, or curative-intent treatment despite imperfect radiologic certainty.

This radiologic–pathologic discordance also underscores the need for standardized pathologic response terminology. Pathologic complete response generally denotes the absence of residual viable tumor in the resected lesion or tumor bed. Major pathologic response denotes a low proportion of residual viable tumor, although the threshold has varied across perioperative HCC studies, including 70% or greater tumor necrosis (i.e., 30% or less residual viable tumor) in some studies and 10% or less residual viable tumor in others. Residual viable tumors should be quantified as the proportion of viable carcinoma across the treated tumor bed, distinguished from necrosis, fibrosis, hemorrhage, and immune-mediated treatment effect, and assessed using a prespecified sampling approach. Future conversion studies should therefore specify how residual viable tumor is measured, whether pathologic response is assessed at the lesion or patient level, and how pathologic response relates to recurrence and survival endpoints.

### 5.3. Molecular and Immune Biomarkers

Molecular and immune biomarkers in HCC have been studied mainly as predictors of response to immune checkpoint inhibition. The detailed mechanistic landscape—including tumor immune microenvironment composition, immune–evasion pathways, and resistance mechanisms—is beyond the scope of this review. Three observations are most relevant to conversion candidacy.

First, immune-inflamed HCC appears to represent a biologically distinct subgroup. An immune class characterized by high immune infiltration was initially described in approximately 25 percent of tumors [76]. Related work defined the broader HCC tumor immune microenvironment [77]. A subsequent integrative analysis refined this classification into inflamed tumors, comprising the original immune subclass and an immune-like subclass, and non-inflamed tumors, comprising intermediate and excluded groups; a 20-gene signature was associated with immunotherapy response [78]. Second, underlying liver disease may influence response. Non-alcoholic steatohepatitis-driven HCC may have impaired antitumor immune surveillance compared with viral-etiology HCC [79]. Progression patterns under atezolizumab plus bevacizumab also appear to differ by liver disease etiology [80]. Third, WNT/CTNNB1-mutated tumors are enriched in the immune-excluded class and tend to respond poorly to ICI-based therapy, consistent with preclinical data linking β-catenin activation to immune escape [78,81].

Several emerging biomarkers may eventually become relevant to conversion decisions, but none is ready for routine candidacy assessment. Intratumoral tertiary lymphoid structures have been associated with reduced early recurrence after HCC resection and may add a structural immune dimension to risk assessment [82]. Circulating tumor DNA and minimal residual disease assessment have demonstrated feasibility in HCC [83]. These approaches may eventually inform post-curative-intent surveillance or reassessment after conversion therapy [84]. Humoral IgG1 responses to tumor antigens have also been linked to clinical outcomes in HCC patients treated with immune checkpoint inhibition [85]. At present, however, these markers should be viewed as research-stage signals. They may help explain response or recurrence risk, but they have not been prospectively validated to determine whether a patient should proceed to resection, ablation, or transplantation.

### 5.4. Physiologic Biomarkers of Surgical Tolerance

Physiologic biomarkers are currently the most actionable biomarkers for conversion candidacy. ALBI grade, sarcopenia indices, and hepatic decompensation history—discussed in detail in Section 4.4—directly inform whether a patient is likely to tolerate the intended curative-intent procedure [63,64,65,66,67,68,69]. Trajectory is as important as the absolute value. Worsening ALBI grade, new ascites, or other signs of hepatic decompensation during treatment should outweigh an otherwise favorable radiographic response. Unlike most molecular biomarkers, physiologic markers do not need to predict immunotherapy response to be clinically useful. Their value lies in identifying patients whose liver reserve or overall condition makes curative-intent treatment unsafe, regardless of how well the tumor has responded.

## 6. Multidisciplinary Workflow for Curative-Intent Transition

**Figure 2:**
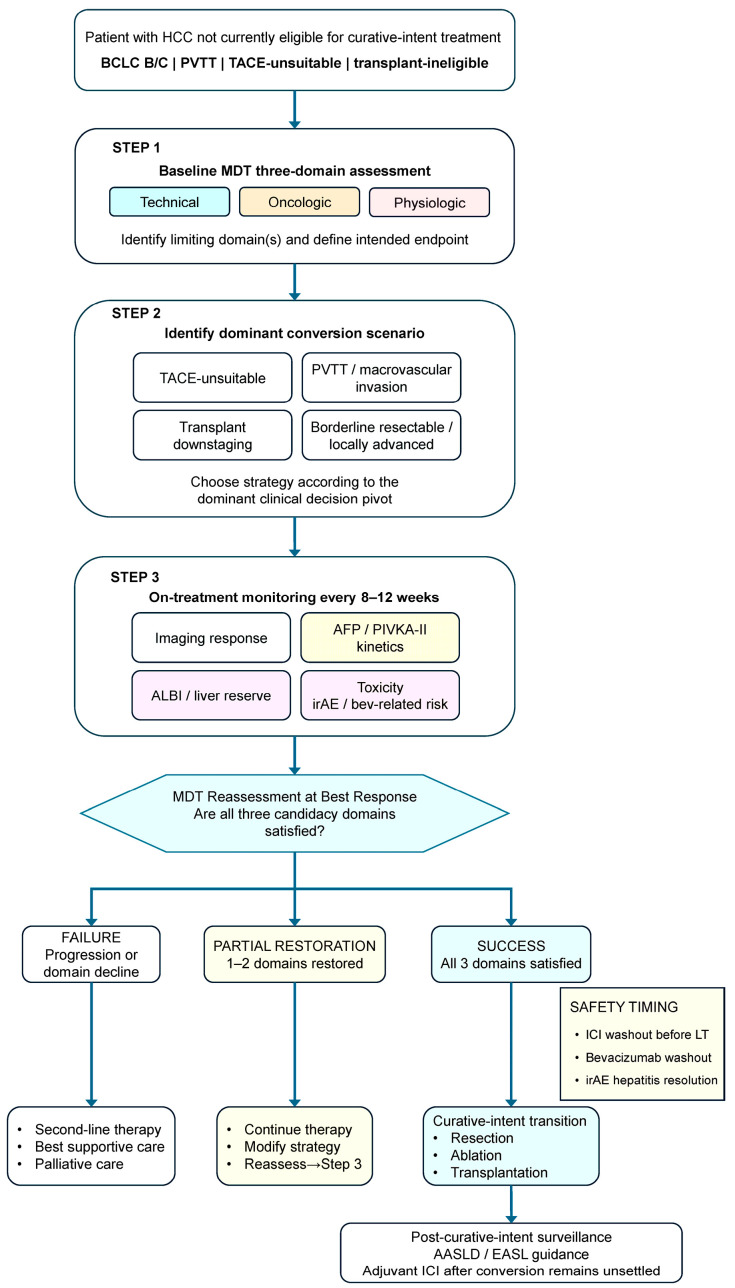
Proposed multidisciplinary workflow for immunotherapy-based conversion to curative-intent treatment in hepatocellular carcinoma. The workflow begins with baseline multidisciplinary assessment of the three candidacy domains and explicit identification of the limiting domain and intended curative-intent endpoint. The dominant conversion scenario is then defined, including TACE-unsuitable disease, portal vein tumor thrombus or macrovascular invasion, transplant downstaging, or borderline/locally advanced resectable disease. During treatment, reassessment every 8 to 12 weeks integrates imaging response, tumor-marker kinetics, liver reserve, and treatment-related toxicity. At best response, multidisciplinary reassessment determines whether candidacy has failed, partially improved, or been restored across all three domains. Patients with insufficient response or domain decline proceed to second-line therapy, best supportive care, or palliative care; patients with partial restoration continue therapy, modify strategy, and undergo repeat reassessment; patients with all three domains satisfied proceed to safety review and curative-intent transition by resection, ablation, or liver transplantation. Post-treatment surveillance follows guideline-based HCC care. Within Steps 1 and 3, blue, yellow, and pink boxes correspond to the technical, oncologic, and physiologic or liver-reserve domains, respectively. In the outcome branch, gray, yellow, and green denote failure, partial restoration, and success, respectively. Arrows indicate the sequential direction of the workflow, branching according to multidisciplinary reassessment, and returning to Step 3 for continued treatment and repeat reassessment after partial restoration. This proposed workflow is intended as a practical decision aid and has not been prospectively validated. Abbreviations: AASLD, American Association for the Study of Liver Diseases; AFP, alpha-fetoprotein; ALBI, albumin-bilirubin; BCLC, Barcelona Clinic Liver Cancer; EASL, European Association for the Study of the Liver; HCC, hepatocellular carcinoma; ICI, immune checkpoint inhibitor; irAE, immune-related adverse event; LT, liver transplantation; MDT, multidisciplinary team; PIVKA-II, protein induced by vitamin K absence or antagonist-II; PVTT, portal vein tumor thrombus; TACE, transarterial chemoembolization.

The three-domain candidacy framework described in Section 4 and the biomarker considerations discussed in Section 5 become clinically useful only when embedded in a multidisciplinary workflow. Curative-intent transition after immunotherapy requires decisions at several timepoints: baseline assessment, on-treatment reassessment, procedural timing, and post-treatment surveillance. Each step involves more than one specialty. The workflow proposed here is summarized in Figure 2 and is based on a simple principle: response data should be interpreted by the same team that will recommend, deliver, or defer the next intervention.

### 6.1. Baseline Multidisciplinary Team Assessment

Multidisciplinary management is a standard component of HCC care and is explicitly recommended in current Western practice guidance [19,86]. Dedicated HCC multidisciplinary clinics have been associated with earlier-stage diagnosis, shorter time to treatment, and improved overall survival compared with non-multidisciplinary care [87]. Multidisciplinary reassessment is also important at recurrence, when treatment options may include repeat resection, ablation, locoregional therapy, or systemic therapy [88]. Recent reviews have incorporated emerging technologies and decision algorithms into this broader HCC multidisciplinary model [89,90]. In parallel, treatment frameworks have moved beyond stage-only allocation toward personalized multiparametric hierarchies that incorporate liver function, tumor biology, and patient preference alongside stage [91]. The updated BCLC framework formalizes treatment stage migration when the standard option is not feasible [59].

For patients being considered for immunotherapy-based conversion, the baseline multidisciplinary team discussion should define two elements before treatment begins: the limiting candidacy domain and the intended curative-intent endpoint. The limiting domain may be technical, such as vascular involvement or inadequate future liver remnants; oncologic, such as high tumor burden, extrahepatic disease, or aggressive marker kinetics; or physiologic, such as portal hypertension, impaired liver reserve, frailty, or immune-related risk. The intended endpoint should also be explicit: resection, ablation, transplantation, or, in selected circumstances, durable treatment-free disease control.

Each specialty contributes a different part of this assessment. The hepatobiliary surgeon evaluates resectability, future liver remnant, and the need for vascular reconstruction. The transplant surgeon and hepatologist assess MELD score, portal hypertension, waitlist feasibility, and post-ICI transplant timing. The medical oncologist selects the systemic regimen, monitors response plateau and immune-related toxicity, and coordinates treatment interruption when local therapy becomes feasible. Interventional radiology and radiation oncology define locoregional or ablative options. Diagnostic radiology and pathology help adjudicate tumor viability and radiologic–pathologic discordance. Without a clearly documented endpoint at baseline, post-treatment reassessment risks becoming reactive rather than goal-directed.

### 6.2. On-Treatment Reassessment

On-treatment reassessment should occur at planned intervals, commonly every 8 to 12 weeks, and should integrate four streams of information: imaging, tumor-marker kinetics, liver function, and treatment-related toxicity. Imaging should be interpreted using both mRECIST and the LI-RADS TR algorithm where applicable [73,74]. Radiologic–pathologic discordance is common after ICI-based therapy and should be kept in mind during interpretation. AFP and PIVKA-II trajectories add a biologic dimension that imaging alone cannot fully capture [22,72]. Liver function should be followed dynamically rather than treated as a baseline variable only. Changes in ALBI grade, new ascites, or encephalopathy may outweigh a favorable scan, particularly because decompensation during therapy can be a stronger driver of mortality than tumor progression in some patients treated with atezolizumab plus bevacizumab [69]. Toxicity surveillance should include irAEs and, for bevacizumab-containing regimens, bleeding risk, hypertension, and proteinuria.

### 6.3. Timing and Safety of Curative-Intent Treatment

The timing of curative-intent treatment after immunotherapy depends on drug pharmacology, immune persistence, and toxicity resolution. For patients proceeding to liver transplantation after ICI exposure, longer washout intervals between the last ICI dose and transplantation have been associated with lower allograft rejection risk in international cohort data [28,29]. For bevacizumab-containing regimens, an interval of at least several weeks is generally needed before major surgery to reduce bleeding and wound-healing risk. Immune-related hepatitis should be fully resolved, with corticosteroids tapered to physiologic dosing when possible, before proceeding to resection or transplantation; prophylactic corticosteroid use to mitigate severe irAEs in HCC has recently been re-examined [92].

Specific washout intervals should not be applied mechanically. The evidence base remains observational, and timings should be individualized according to the intended procedure, degree of response, residual toxicity, liver reserve, and transplant urgency. A recent transplant-focused expert consensus is consistent with this individualized approach, suggesting a minimum ICI washout of 30 to 50 days—and approximately 90 days when feasible—together with at least 3 months of radiographic stability after downstaging before transplantation [93]. A multidisciplinary clinic structure has been associated with more consistent on-treatment monitoring, longer time on therapy, and fewer liver-related treatment discontinuations in unresectable HCC, although without a significant overall survival benefit [94]. Real-world cohorts are also beginning to define therapeutic sequences after atezolizumab plus bevacizumab, providing practical guidance for decision-making at progression or response plateau [95].

### 6.4. Curative-Intent Endpoints and Post-Treatment Surveillance

Curative-intent endpoints after immunotherapy include resection, ablation, and liver transplantation. Sustained treatment-free disease control, discussed in Section 2 and Section 3.1, is an additional emerging endpoint for selected patients who do not undergo local therapy, but it should not be equated with anatomic cure. After curative-intent treatment, surveillance should follow current HCC practice guidance, including that from American Association for the Study of Liver Diseases and European Association for the Study of the Liver [19,86], with particular attention to liver function trajectory in patients who experienced decompensation during systemic therapy.

Whether adjuvant immunotherapy improves outcomes after curative-intent surgery remains unsettled. The IMbrave050 phase 3 trial of adjuvant atezolizumab plus bevacizumab in resected high-risk HCC initially reported a recurrence-free survival benefit [51]. On updated analysis, however, the recurrence-free survival benefit was not maintained, and overall survival showed a trend toward harm while remaining immature [52]. Adjuvant ICI after conversion surgery should therefore be approached cautiously and, when possible, within a clinical trial framework until more mature data are available.

## 7. Comparative Lessons from Gastrointestinal Oncology

The framework proposed in this review is HCC-specific, but it is informed by experience in adjacent gastrointestinal malignancies. Pancreatic adenocarcinoma, biliary tract cancer, and colorectal liver metastases (CRLM) each face a related problem: when should response to systemic therapy justify a curative-intent operation? The answer differs by disease. Pancreatic cancer offers the logic of multidomain candidacy, biliary tract cancer illustrates the limits of downstaging in biologically aggressive disease, and CRLM provide the most mature example of prospectively defined conversion endpoints and multidisciplinary reassessment.

### 7.1. Pancreatic Borderline Resectability

The borderline-resectable framework for pancreatic adenocarcinoma is the most mature multidomain candidacy construct in gastrointestinal surgical oncology. As discussed in Section 4.1, its anatomic, biologic, and conditional triad was operationalized in 2008, formalized by the National Comprehensive Cancer Network, and later supported as a basis for neoadjuvant therapy by the PREOPANC trial [54,55,56]. The pancreatic experience demonstrates two transferable principles: candidacy can be defined along more than one axis without losing clinical usefulness, and neoadjuvant therapy can function as both treatment and selection.

### 7.2. Biliary Tract Cancer and Intrahepatic Cholangiocarcinoma

Biliary tract cancer has moved more slowly toward systemic downstaging and conversion. After gemcitabine plus cisplatin became the systemic backbone, immune checkpoint inhibition added to this regimen improved overall survival in advanced disease, as shown in TOPAZ-1 and KEYNOTE-966 [96,97,98]. For intrahepatic cholangiocarcinoma, an oncological resectability framework adapted from solid-organ surgical oncology has recently been proposed, paralleling the three-domain logic developed here, although the conversion evidence base remains thinner and retrospective [99,100]. The lesson is therefore cautionary: biologic aggressiveness and early systemic failure may limit the degree to which radiographic response translates into durable curative-intent benefit.

### 7.3. Colorectal Liver Metastases

CRLM offers the most mature conversion paradigm in gastrointestinal surgical oncology. Initially unresectable CRLM can be rendered resectable through chemotherapy, with rescue surgery associated with long-term survival in selected responders; intensified regimens such as FOLFOXIRI plus bevacizumab have further improved conversion to resection [101,102]. Three lessons from CRLM are directly relevant to HCC. First, dynamic reassessment at planned intervals, rather than a single post-treatment scan, is essential when conversion is the intent. Second, response to systemic therapy functions as a biological test that supplements anatomic resectability assessment. Third, multidisciplinary team management, rather than serial single-specialty consultation, is the operational structure that has made conversion routine in CRLM practice [103,104].

### 7.4. What HCC Can Adapt—And What It Cannot

These gastrointestinal parallels suggest that HCC can adopt several principles: multidomain candidacy assessment, planned reassessment during systemic therapy, and multidisciplinary ownership of the conversion decision. What HCC cannot import directly is the assumption of a healthy substrate organ. Cirrhosis, clinically significant portal hypertension, hepatic reserve, transplant allocation, and the differing biology of viral, alcohol-related, and metabolic dysfunction-associated steatotic liver disease impose constraints that pancreatic and colorectal conversion frameworks do not address. The HCC-specific framework proposed in Section 4 therefore shares conceptual roots with the pancreatic anatomic, biologic, and conditional axes, but is an adaptation rather than a direct transfer of that model. The distinction matters because, in HCC, the underlying liver is itself part of the disease.

## 8. Conclusions and Future Directions

Immunotherapy-based combinations have created new opportunities for selected patients with HCC to undergo curative-intent treatment after initially non-curative-intent presentations. However, the evidence base for conversion remains heterogeneous, and conversion-relevant endpoints—pathologic response, transition to resection or transplantation, and durable treatment-free status—are inconsistently defined across trials, regions, and specialties. Radiographic response alone is therefore insufficient to determine whether a patient should proceed to curative-intent treatment.

This review proposes a multidisciplinary framework in which curative-intent transition is defined by the intersection of three suitability domains: technical, oncologic, and physiologic or liver-reserve. Liver transplantation is treated as a curative-intent pathway requiring all three domains rather than as a separate fourth domain. The framework is informed by the borderline-resectable construct in pancreatic adenocarcinoma, but adapted to HCC-specific constraints, including cirrhosis, portal hypertension, hepatic reserve, transplant allocation, and the differing biology of viral, alcohol-related, and metabolic dysfunction-associated steatotic liver disease.

Several priorities should shape the next phase of research and clinical practice. Prospective conversion registries with prespecified curative-intent endpoints are needed to move the field beyond retrospective case series. Candidate biomarkers—including AFP and PIVKA-II kinetics, immunogenomic class taxonomy, and physiologic measures such as ALBI grade and sarcopenia—should be evaluated specifically as candidacy biomarkers rather than only as response biomarkers. The terminology divergence between Asian conversion-anchored and Western downstaging-anchored frameworks should be harmonized through shared lexicon and endpoint definitions. Safety data on liver transplantation after ICI exposure are emerging, but remain insufficient to standardize washout intervals or immunosuppression strategy. Prospective collection of these data is a near-term priority. The role of adjuvant immunotherapy after curative-intent surgery also remains controversial in light of the updated IMbrave050 overall survival analysis [52]. It should be defined through trials with appropriate post-conversion endpoints. Finally, the multidisciplinary workflow proposed here should be evaluated for its impact on conversion rate, post-conversion survival, liver-related morbidity, and patient-reported outcomes in real-world practice.

The central message is practical. Immunotherapy expands the population in whom curative-intent treatment can be reconsidered, but it does not replace the criteria by which curative-intent treatment should be offered. A response-only approach may miss some patients who could benefit while exposing others to procedures unlikely to provide durable benefit. Because this framework is based on expert opinion and has not been prospectively validated, it should be viewed as a structure for multidisciplinary decision-making and future study design rather than as formal selection criteria. Applied in this way, a multidomain candidacy framework and multidisciplinary team workflow may provide a more reproducible way to translate systemic therapy progress into durable curative-intent outcomes for patients with HCC.

## Figures and Tables

**Table 1:** Lexicon of curative-intent transition terms in hepatocellular carcinoma.

Term	Practical Definition	Typical Starting Scenario	Intended Endpoint	Regional or Specialty Nuance	Common Pitfall
Conversion therapy	Systemic and/or locoregional therapy intended to render initially non-curative-intent HCC eligible for resection, ablation, or transplantation [16,17,18].	Initially unresectable, borderline-resectable, or TACE-unsuitable HCC, including selected patients with BCLC B/C disease or PVTT.	Usually resection, particularly in Asian practice; selected patients may proceed to ablation or transplantation.	Most prominent in Asian resection-anchored literature; increasingly adopted in Western literature.	Often conflated with downstaging or neoadjuvant therapy. The intended curative endpoint should be defined at baseline whenever possible.
Downstaging	Reduction in tumor burden from beyond predefined transplant eligibility criteria to within acceptable criteria for liver transplantation [19,20,21,22].	HCC exceeding Milan, UCSF, UNOS downstaging, or AFP-based transplant eligibility frameworks.	Liver transplantation.	Western transplant-anchored concept; often protocol-defined in waitlist systems.	Requires predefined baseline and reassessment criteria. Ad hoc tumor shrinkage is not equivalent to protocol-based downstaging.
Bridging therapy	Usually locoregional, and occasionally systemic, therapy used to prevent tumor progression while awaiting liver transplantation [19,23].	HCC within accepted transplant criteria in patients listed, or being evaluated, for liver transplantation.	Liver transplantation while maintaining eligibility.	Primarily a Western waitlist concept; treatment modalities overlap with those used for downstaging.	Distinct from downstaging: bridging maintains eligibility, whereas downstaging restores eligibility.
Neoadjuvant therapy	Systemic and/or locoregional therapy given before planned curative-intent treatment in patients already eligible at baseline [24,25,26,27].	Resectable HCC with high-risk, borderline technical, or high-recurrence-risk features.	Most commonly resection; occasionally ablation or transplantation depending on pathway.	Emerging mainly in Western trial frameworks; conceptually analogous to borderline-resectable pancreatic cancer.	Distinct from conversion therapy because patients are already curative-intent candidates at baseline.
Post-ICI transplantation/ICI bridge to transplantation	ICI-based therapy before planned or possible liver transplantation, followed by reassessment of transplant candidacy, washout interval, and rejection risk [23,28,29].	HCC patients exposed to ICI-based therapy, who are being considered for liver transplantation.	Liver transplantation.	Rapidly evolving transplant-oncology area; safety and optimal washout interval remain unsettled.	Drug half-life does not fully capture immune persistence. Washout interval, prior immune-related toxicity, and immunosuppression strategy should be considered.
Drug-free or treatment-free status	Sustained complete or near-complete response after stopping systemic therapy without immediate resection or liver transplantation [30].	Complete or near-complete response after ICI-based combination therapy, often in patients initially unsuitable for curative-intent local therapy.	Durable disease control without ongoing systemic therapy; does not necessarily imply anatomic cure.	Increasingly discussed in Asian frameworks after atezolizumab–bevacizumab-based deep responses.	Should not replace feasible curative-intent local therapy. Long-term durability remains uncertain.

This table provides practical definitions for multidisciplinary communication rather than formal regulatory definitions. Terms may overlap in clinical practice, but they differ by baseline candidacy, intended endpoint, and regional or specialty context. AFP, alpha-fetoprotein; BCLC, Barcelona Clinic Liver Cancer; HCC, hepatocellular carcinoma; ICI, immune checkpoint inhibitor; PVTT, portal vein tumor thrombus; TACE, transarterial chemoembolization; UCSF, University of California San Francisco; UNOS, United Network for Organ Sharing.

**Table 2:** Representative evidence for immunotherapy-based conversion strategies in hepatocellular carcinoma, by clinical decision setting and evidence certainty.

Clinical Decision Setting	Representative Strategy	Representative Evidence	Conversion-Relevant Signal	Evidence Certainty for Conversion Decisions	Practical Interpretation/Main Limitation
Systemic backbone for advanced HCC	ICI-VEGF combination (atezolizumab plus bevacizumab)	IMbrave150 (Finn et al. 2020) [7]	ORR of approximately 30% with durable responses established atezolizumab plus bevacizumab as a major first-line systemic backbone.	High for systemic disease control; lower for conversion decisions	Substrate for many reported conversion case series; pivotal trial was not designed with curative-intent conversion as an endpoint.
Systemic backbone for advanced HCC	Dual immune checkpoint blockade	HIMALAYA (Abou-Alfa et al. 2022) [8]; CheckMate 9DW (Yau et al. 2025) [9]	Durable responses with long-term survival tails; CheckMate 9DW demonstrated a high ORR among phase 3 ICI-based regimens.	High for systemic disease control; lower for conversion decisions	Bevacizumab-free regimens may be useful in patients with bleeding risk; early toxicity and physiologic selection remain important.
Systemic backbone for advanced HCC	ICI-TKI/ICI-VEGFR combinations	CARES-310 (Qin et al. 2025) [10]; LEAP-002 (Llovet et al. 2023) [11]; COSMIC-312 (Yau et al. 2024) [12]	CARES-310 supports an Asia-led ICI-TKI approach; LEAP-002 and COSMIC-312 did not improve OS despite PFS or response signals.	High for systemic disease control; lower for conversion decisions	Combinations should not be assumed equivalent. ORR or PFS gains do not consistently translate into OS benefit in HCC.
TACE-unsuitable or intermediate-stage HCC	TACE combined with immunotherapy-based systemic therapy	EMERALD-1 (Sangro et al. 2025) [13]; LEAP-012 (Kudo et al. 2025) [14]; Singal 2026 commentary [39]	Phase 3 trials showed improved PFS with systemic integration into TACE-based treatment.	High for PFS in intermediate-stage disease; lower for conversion decisions	Supports earlier systemic integration in BCLC-B disease, but conversion to resection, ablation, or liver transplantation was not the primary endpoint.
TACE-unsuitable or intermediate-stage HCC	ICI plus TKI plus TACE in selected TACE-unsuitable patients	Chen et al. 2024 [40]	Early-phase data reported substantial conversion-to-resection rates in selected responders.	Low; prospective single-arm, selected conversion cohort	Supports the Asian conversion paradigm; evidence remains highly selected and requires broader validation.
Deep response without immediate local therapy	Atezolizumab plus bevacizumab followed by treatment discontinuation	Kudo et al. 2023 drug-free status [30]	Complete or near-complete responders may maintain treatment-free status after stopping systemic therapy.	Low; proof-of-concept cohort	Relevant when curative-intent local therapy is not feasible; treatment-free status should not be equated with anatomic cure.
Macrovascular invasion/PVTT	HAIC-based or multimodal locoregional-systemic therapy for high-burden or PVTT disease	FOHAIC-1 (Lyu et al. 2022) [41]; Cai et al. 2024 [42]	HAIC improved OS versus sorafenib and enabled curative surgery or ablation in a subset; HAIC added to lenvatinib plus DEB-TACE improved response and survival in major PVTT.	Moderate for disease control; lower for conversion decisions	Important Asia-Pacific strategy for PVTT-heavy disease; applicability depends on institutional expertise and immunotherapy-specific conversion endpoints remain limited.
Macrovascular invasion/PVTT	Radiation-based integration with systemic therapy	RTOG 1112 (Dawson et al. 2024) [43]; PEMRAD (O’Kane et al. 2025) [44]	SBRT-based approaches improved local control or response, including in macrovascular invasion.	Moderate for local control or survival; lower for conversion decisions	Supports local control as part of conversion strategy; immunotherapy-specific phase 3 conversion data remain limited.
Macrovascular invasion/PVTT	Preoperative lenvatinib for Vp3 or Vp4 disease	Ichida et al. 2024 [45]	Preoperative lenvatinib maintained surgical feasibility and yielded R0 resection and favorable 1-year survival in selected patients.	Low; prospective single-arm, selected surgical cohort	Reinforces the resection-anchored Asian paradigm; broader validation is needed.
Macrovascular invasion/PVTT	Systemic ICI-TKI conversion followed by resection	SILENSES (Lu et al. 2026) [46]	Prospective trial with conversion rate as the primary endpoint: 56% successful conversion, 60 resections; 5-year OS 73.9% in the surgical cohort.	Low to moderate; prospective single-center, single-arm conversion study	Direct prospective conversion signal; single-center, single-arm, highly selected, predominantly HBV/Asian.
Borderline-resectable or locally advanced HCC	Neoadjuvant or perioperative ICI	Marron et al. 2022 [26]; Kaseb et al. 2022 [25]; Lin et al. 2026 [47]	Pathologic responses after ICI-based therapy support neoadjuvant immunotherapy as a selection and treatment strategy.	Low to moderate; small prospective perioperative studies	Pathologic response may be more informative than radiographic response; optimal regimen and timing remain unsettled.
Borderline-resectable or locally advanced HCC	ICI combined with TKI before surgery	Ho et al. 2021 [24]	Cabozantinib plus nivolumab enabled R0 resection in many patients within a small proof-of-concept cohort.	Very low; small proof-of-concept cohort	Strong conversion signal; sample size is small and patient selection is critical.
Transplant downstaging or bridging	ICI exposure before liver transplantation	VITALITY (Tabrizian et al. 2025) [23]; Aceituno et al. 2026 [29]	Multicenter data suggest that transplantation after ICI exposure is feasible in selected patients.	Low to moderate; retrospective or pooled transplant cohorts	Feasibility is increasingly supported; rejection risk and selection bias remain major concerns.
Transplant downstaging or bridging	ICI washout before liver transplantation	Moeckli et al. 2025 [28]	Longer washout interval was associated with lower rejection risk after transplantation.	Low; retrospective cohort	Provides practical sequencing guidance for MDT decisions; the optimal washout interval is not yet standardized.

Representative evidence is organized by clinical decision setting rather than as a comprehensive trial inventory. The table includes phase 3 randomized trials, selected phase 2 studies, and clinically influential cohorts that inform conversion-relevant decision-making. Conversion-relevant signals include objective response, pathologic response, durable treatment-free response, and transition to resection, ablation, or liver transplantation. Trial-level design, population, and efficacy detail are provided in Supplementary Table S1. Evidence certainty refers to the ability of the cited evidence to inform curative-intent conversion decisions, not necessarily to the certainty of systemic disease-control benefit. Certainty was assessed qualitatively for this narrative review; no formal grading instrument, such as GRADE, was applied. BCLC, Barcelona Clinic Liver Cancer; DEB-TACE, drug-eluting bead transarterial chemoembolization; HAIC, hepatic arterial infusion chemotherapy; HBV, hepatitis B virus; HCC, hepatocellular carcinoma; ICI, immune checkpoint inhibitor; MDT, multidisciplinary team; ORR, objective response rate; OS, overall survival; PFS, progression-free survival; PVTT, portal vein tumor thrombus; R0, margin-negative resection; SBRT, stereotactic body radiotherapy; TACE, transarterial chemoembolization; TKI, tyrosine kinase inhibitor; VEGF, vascular endothelial growth factor; VEGFR, vascular endothelial growth factor receptor; Vp3/Vp4, portal vein invasion class 3/4.

## Data Availability

No new data were created or analyzed in this study. Data sharing is not applicable to this article.

## Supplementary Materials

The following supporting information can be downloaded at: https://www.mdpi.com/article/10.3390/cancers18142234/s1, Table S1: Trial-level evidence supporting immunotherapy-based conversion strategies in hepatocellular carcinoma.

## Abbreviations

The following abbreviations are used in this manuscript:

AFP	Alpha-fetoprotein
ALBI	Albumin-bilirubin
BCLC	Barcelona Clinic Liver Cancer
CRLM	Colorectal liver metastases
HAIC	Hepatic arterial infusion chemotherapy
HCC	Hepatocellular carcinoma
ICI	Immune checkpoint inhibitor
irAE	Immune-related adverse event
LI-RADS TR	Liver Imaging Reporting and Data System Treatment Response
MELD	Model for End-Stage Liver Disease
mRECIST	Modified Response Evaluation Criteria in Solid Tumors
PIVKA-II	Protein induced by vitamin K absence or antagonist-II
PVTT	Portal vein tumor thrombus
RETREAT	Risk Estimation of Tumor Recurrence After Transplant score
R0	Margin-negative resection
SBRT	Stereotactic body radiotherapy
TACE	Transarterial chemoembolization
UCSF	University of California San Francisco
UNOS	United Network for Organ Sharing
Vp3/Vp4	Portal vein invasion class 3/4
