# Supplementary material for: Immunotherapy-Based Conversion to Curative-Intent Treatment in Hepatocellular Carcinoma: A Multidisciplinary Framework
Source: Cancers (Basel). 2026 Jul 12;18(14):2234. doi: 10.3390/cancers18142234 (PMC13406758; doi:10.3390/cancers18142234)
Supplement: Supplementary file 1 [file cancers-18-02234-s001.zip › cancers-4408025-supplementary.pdf]

cancers MDPI

Supplementary Table S1. Trial-level evidence supporting immunotherapy-based conversion strategies in hepatocellular carcinoma

Trial / study	Design / population	Key efficacy	Conversion-relevant signal	Main limitation
=== A. Systemic backbone ===				
SHARP, Llovet et al. 2008 (NCT00105443) [3]	Phase 3 RCT; sorafenib vs placebo; advanced HCC, Child-Pugh A	OS 10.7 vs 7.9 mo; HR 0.69	ORR 2%; minimal conversion potential	Historical comparator
REFLECT, Kudo et al. 2018 (NCT01761266) [4]	Phase 3 non-inferiority; lenvatinib vs sorafenib; advanced HCC	OS 13.6 vs 12.3 mo; HR 0.92	ORR 18.8% vs 6.5% (RECIST 1.1)	Not designed for conversion endpoints
IMbrave150, Finn et al. 2020 (NCT03434379) [7]	Phase 3 RCT; atezolizumab + bevacizumab vs sorafenib; unresectable HCC	OS HR 0.58	ORR ~30% with durable responses	Conversion not a primary endpoint
HIMALAYA, Abou-Alfa et al. 2022 (NCT03298451) [8]	Phase 3 RCT; tremelimumab + durvalumab vs sorafenib; unresectable HCC	OS 16.4 vs 13.8 mo; HR 0.78	Durable responses; bevacizumab-free	Conversion data limited
CheckMate 9DW, Yau et al. 2025 (NCT04039607) [9]	Phase 3 RCT; nivolumab + ipilimumab vs lenvatinib/sorafenib; unresectable HCC, CP 5-6	OS 23.7 vs 20.6 mo; HR 0.79; ORR 36% vs 13%	High ORR among phase 3 ICI-based regimens	Early toxicity; physiologic selection critical
CARES-310 final, Qin et al. 2025 (NCT03764293) [10]	Phase 3 RCT; camrelizumab + rivoceranib vs sorafenib; unresectable HCC, predominantly Asian	OS 23.8 vs 15.2 mo; HR 0.64; PFS HR 0.54	Asia-led ICI-TKI platform	Regional generalizability limited
LEAP-002, Llovet et al. 2023 (NCT03713593) [11]	Phase 3 RCT; lenvatinib + pembrolizumab vs lenvatinib; advanced HCC	OS 21.2 vs 19.0 mo; HR 0.84 (NS)	Numerical activity; no OS benefit	Active TKI comparator dilutes signal
COSMIC-312 final, Yau et al. 2024 (NCT03755791) [12]	Phase 3 RCT; cabozantinib + atezolizumab vs sorafenib; advanced HCC	OS HR 0.98; PFS HR 0.74	PFS benefit without OS translation	PFS imperfect surrogate in HCC
=== B. TACE-unsuitable / intermediate-stage HCC (includes deep response) ===				
EMERALD-1, Sangro et al. 2025 (NCT03778957) [13]	Phase 3 RCT; TACE + durvalumab ± bevacizumab vs TACE; TACE-eligible intermediate-stage HCC	PFS HR 0.77 (durvalumab + bevacizumab + TACE vs TACE)	Supports systemic integration with TACE	Conversion not a primary endpoint

 https://doi.org/10.3390/cancers18142234

LEAP-012, Kudo et al. 2025 (NCT04246177) [14]	Phase 3 RCT; TACE + lenvatinib + pembrolizumab vs TACE; intermediate-stage HCC, TACE-eligible	PFS HR 0.66; median OS not reached	Curative-intent reassessment in selected responders	Conversion not formally tested
Chen et al. 2024 [40]	Prospective single-arm phase 2; envafolimab + lenvatinib + TACE; TACE-unsuitable unresectable HCC	Surgical conversion 47.2% (17/36); 16 R0 resections	Conversion to resection in selected responders	Single-arm; small; highly selected
Kudo et al. 2023 drug-free status cohort [30]	Multicenter proof-of-concept; complete or near-complete responders to atezolizumab + bevacizumab	Drug-free status >12 mo in a subset	Treatment-free disease control after deep response	Not equivalent to cure; durability uncertain
=== C. Macrovascular invasion / PVTT ===				
FOHAIC-1, Lyu et al. 2022 (NCT03164382) [41]	Phase 3 RCT; HAIC-FO vs sorafenib; advanced HCC with high burden or Vp3/Vp4 PVTT	OS 13.9 vs 8.2 mo; HR 0.41	12.3% downstaged; curative surgery or ablation in a subset	HAIC expertise required; Asia-Pacific context
Cai et al. 2024 [42]	Multicenter retrospective; HCC >7 cm with major PVTT; lenvatinib + DEB-TACE ± HAIC	ORR 61% vs 34%; OS 16.7 vs 12.5 mo	Added HAIC improved response and survival in major PVTT	Retrospective; conversion not an endpoint
RTOG 1112, Dawson et al. 2024 (NCT01730937) [43]	Phase 3 RCT; SBRT + sorafenib vs sorafenib; advanced HCC unsuitable for or refractory to standard therapies	OS 15.8 vs 12.3 mo; HR 0.77	Improved local control; OS benefit significant after adjustment for stratification factors	Not immunotherapy-based
PEMRAD, O'Kane et al. 2025 [44]	Phase 2; SBRT + pembrolizumab; post-sorafenib advanced HCC	ORR 31%; median OS 11.1 mo	Response in technically challenging disease	Phase 3 lacking
Ichida et al. 2024 [45]	Multicenter phase 2; preoperative lenvatinib; advanced HCC planned for hepatectomy	R0 82%; 1-yr OS 76%	Surgical feasibility after preoperative TKI	External validation needed
SILENSES, Lu et al. 2026 (ChiCTR1900023914) [46]	Prospective single-arm phase 2 expansion; sintilimab + lenvatinib; initially unresectable HCC, predominantly BCLC-C with macrovascular invasion	Conversion 56% (67/120); ORR 58% (mRECIST); surgical cohort 5-yr OS 73.9%, median RFS 40 mo; pCR 35% (21/60)	Conversion rate as primary endpoint; 60 resections after MDT assessment	Single-center, single-arm; highly selected; predominantly HBV/Asian

=== D. Borderline resectable or locally advanced ===				
Marron et al. 2022 [26]	Phase 2; neoadjuvant cemiplimab; resectable HCC	Major pathologic response 20%	Pathologic response supports neoadjuvant ICI	Conversion relevance indirect
Kaseb et al. 2022 [25]	Phase 2; perioperative nivolumab vs nivolumab + ipilimumab; resectable HCC	Major pathologic response 33% (3/9) with nivolumab vs 27% (3/11) with nivolumab + ipilimumab; pathologic complete responses in both arms	Pathologic responses with perioperative ICI; no clear superiority of dual over monotherapy	Small study; optimal regimen uncertain
Ho et al. 2021 [24]	Phase 1b; cabozantinib + nivolumab; locally advanced HCC	12/15 patients underwent R0 resection	Proof-of-concept conversion signal	Small, highly selected cohort
Lin et al. 2026 [47]	Prospective phase 2 follow-up with biomarker analysis; potentially resectable HCC	Long-term efficacy and biomarker data	Supports dual-ICI perioperative platform	Closely related to prior studies
=== E. Transplant downstaging / bridging ===				
VITALITY, Tabrizian et al. 2025 [23]	Multicenter intention-to-treat cohort; waitlisted HCC receiving ICI as bridging or downstaging	3-yr ITT 71.1%; post-LT 85%; 36.7% transplanted	Feasibility supported at scale	Selection bias; center expertise critical
Moeckli et al. 2025 [28]	International retrospective cohort; 119 HCC patients receiving ICI before liver transplantation	Rejection 20%; washout >50 days reduces rejection	Practical washout guidance	Washout interval not standardized
Aceituno et al. 2026 [29]	Pooled cohort and meta-analysis; ICI recipients before liver transplantation	Rejection ~15-20%, varies by washout	Complementary safety evidence	Heterogeneous treatment/timing
=== F. Adjacent context ===				
IMbrave050, Qin et al. 2023 [51]; Yopp et al. 2026 update [52]	Phase 3 RCT; adjuvant atezolizumab + bevacizumab; resected high-risk HCC	Initial RFS HR 0.72; updated RFS HR 0.90 (not maintained), OS HR 1.26 (immature)	Informs post-conversion adjuvant sequencing	Not conversion; benefit not sustained
HOPE4LIVER, Mendiratta-Lala et al. 2024 (NCT04572633 / NCT04573881) [53]	Single-arm pivotal histotripsy trial; primary or metastatic liver tumors <3 cm	Technical success 95%; major complications 7%	Bridge to liver transplantation or resection in select anatomy	Non-immunotherapy modality; included as adjacent bridging context

This supplementary table provides trial-level detail supporting Table 2 in the main text. Rows are grouped by the same clinical decision settings as main Table 2 (sections A through E) and include representative phase 3 randomized trials, phase 2 studies, and clinically influential cohorts. Section F includes adjacent-context studies that inform the manuscript discussion but are not strict immunotherapy-based conversion evidence: IMbrave050 with its Yopp 2026 update informs adjuvant

https://doi.org/10.3390/cancers18142234

sequencing after curative-intent transition, whereas HOPE4LIVER informs non-immunotherapy bridging options. Reference numbers in this table correspond to the numbering in the main manuscript reference list.

Abbreviations: CP, Child-Pugh; CR, complete response; DEB-TACE, drug-eluting bead transarterial chemoembolization; HAIC, hepatic arterial infusion chemotherapy; HAIC-FO, HAIC with fluorouracil + oxaliplatin; HCC, hepatocellular carcinoma; HR, hazard ratio; ICI, immune checkpoint inhibitor; ITT, intention-to-treat; LT, liver transplantation; mo, months; NCT, ClinicalTrials.gov identifier; NS, not statistically significant; ORR, objective response rate; OS, overall survival; pCR, pathological complete response; PFS, progression-free survival; PVTT, portal vein tumor thrombus; R0, margin-negative resection; RCT, randomized controlled trial; RECIST 1.1, Response Evaluation Criteria in Solid Tumors version 1.1; RFS, recurrence-free survival; SBRT, stereotactic body radiotherapy; TACE, transarterial chemoembolization; TKI, tyrosine kinase inhibitor; Vp3/Vp4, portal vein invasion class 3/4.

https://doi.org/10.3390/cancers18142234
